# Progress and applications of mass cytometry in sketching immune landscapes

**DOI:** 10.1002/ctm2.206

**Published:** 2020-10-14

**Authors:** Ting Zhang, Antony R. Warden, Yiyang Li, Xianting Ding

**Affiliations:** ^1^ State Key laboratory of Oncogenes and Related Genes, Institute for Personalized Medicine, School of Biomedical Engineering Shanghai Jiao Tong University Shanghai China

**Keywords:** immune landscape, mass cytometry, proteome, single‐cell

## Abstract

Recently emerged mass cytometry (cytometry by time‐of‐flight [CyTOF]) technology permits the identification and quantification of inherently diverse cellular systems, and the simultaneous measurement of functional attributes at the single‐cell resolution. By virtue of its multiplex ability with limited need for compensation, CyTOF has led a critical role in immunological research fields. Here, we present an overview of CyTOF, including the introduction of CyTOF principle and advantages that make it a standalone tool in deciphering immune mysteries. We then discuss the functional assays, introduce the bioinformatics to interpret the data yield via CyTOF, and depict the emerging clinical and research applications of CyTOF technology in sketching immune landscape in a wide variety of diseases.

## Graphical abstract

1. The CyTOF technique enables immune profiling of various disease conditions.

2. The development of CyTOF‐based functional assays and bioinformatics pipelines is thriving and promising.

3. CyTOF may serve as a promising platform to facilitate the identification of immunological biomarkers and drug screening.

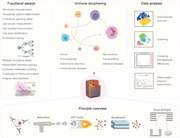

## 1 INTRODUCTION

The immune plasticity is expressed in the remarkably heterogeneous milieus, including circulation and different tissues, during the homeostasis or during the various disease states.^1^ An array of adaptive responses, including training, priming, exhaustion, and tolerance, has been distinguished in adaptive and innate immunocytes. Precise classification of cell subpopulations with overlapping phenotypes and simultaneous interrogation of the phenotypic and functional properties of single cells in basal state and after their exposure to exogenous stimuli are of great significance.

Fluorescence‐based cytometry techniques have dominated for decades in immune system studies at the single‐cell resolution. However, the number of parameters available and complex compensation processes for spectral overlap limit its applications. The developments of fluorescence‐based cytometry have been propelled by recent technological advances. Notably, cytometry by time‐of‐flight (CyTOF), also known as mass cytometry, is a novel combination of flow cytometry and mass spectrometry, which excels in multiparametric single‐cell analysis. CyTOF, through the utility of rare‐earth metal‐tagged antibodies, inductively coupled plasma ionization, and time‐of‐flight detector, allows simultaneous characterization of up to 50 parameters per cell. Therefore, CyTOF provides significant possibilities toward the identification of disease attributes in cell populations, the orchestrated interplay amongst protean immune cells, as well as the molecular immunological signatures that underlie clinical manifestations.

Here, we briefly introduce the principle, advantages, and current limitations of CyTOF. We then describe current progresses in functional assay developments leveraging CyTOF and the developed bioinformatic techniques and pipelines in CyTOF data analysis. Next, we discuss recent applications of CyTOF in basic and clinical research of immune profiling in the fields of cancer, immunotherapy, autoimmune diseases, infective diseases, cardiovascular diseases, transplantation, and neuroscience. Finally, we comment on the perspectives of CyTOF's rapid entry into research and clinical settings.

## 2 OVERVIEW OF CyTOF's WORKING PRINCIPLES

**FIGURE 1:**
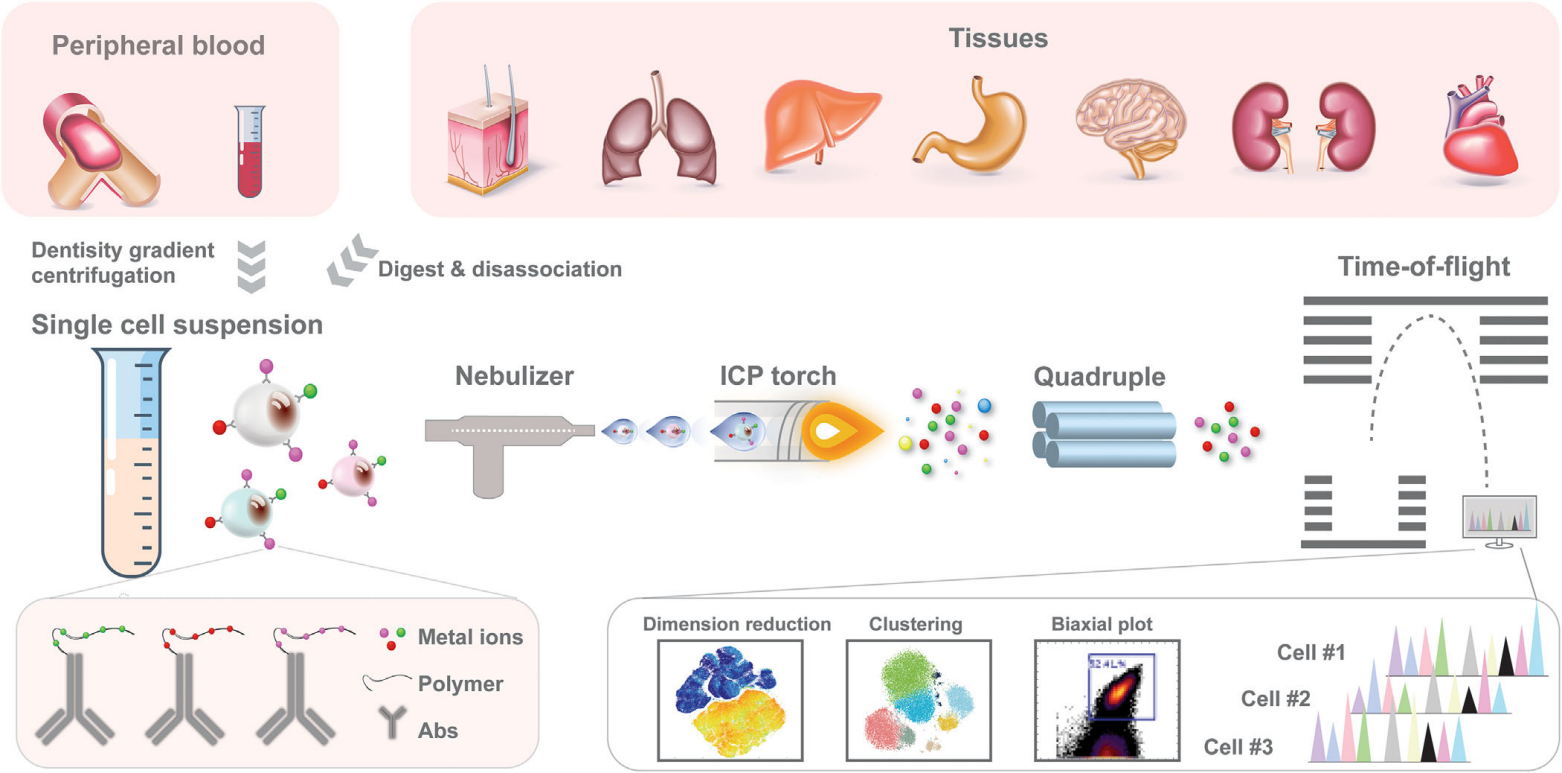
Overview of CyTOF analysis

The technical aspects of the CyTOF system have been described in detail by Bendall et al,^2^ as depicted in Figure 1. In essence, instead of fluorophores, antibodies are labeled with stable heavy metal isotopes, mainly of the lanthanide series, which are naturally absent in biological systems. Each isotope's readout can be correlated with a specific antibody probe, representing the antigen levels within individual cells. Sample preparation and staining for CyTOF are similar to fluorescence flow cytometry, except that cells are typically fixed prior to analysis. Cells are then introduced into the CyTOF analyzer and nebulized into droplets, which are vaporized, atomized, ionized, and then accelerated toward a mass spectrometer via electrical potential. The current time‐of‐flight (TOF) detector of commercialized CyTOF is tuned for a mass window of approximately 89‐209 Da, and the remaining atoms are filtered by a quadrupole to increase sensitivity and minimize unwanted signals. The filtered ion clouds are analyzed with TOF detector.

## 3 ADVANTAGES AND LIMITATIONS OF CyTOF

rDespite being generally considered an alteration based on fluorescence flow cytometry, mass cytometry differs from fluorescence cytometry by several aspects including dimensionality, sensitivity, cell throughput, and so on (Table 1). The clever switch from fluorescence readout to mass spectrometry detection of heavy metal‐tagged antibody probes has given CyTOF several unique advantages. First, the increment in dimensionality represents a groundbreaking breakthrough. In fluorescence flow cytometry, the number of detection parameters is limited by the overlapping of fluorescence signals. This problem is especially prominent when performing antigen‐specific T cells screening, chromatin modification profiling, and simultaneous detection of RNAs and proteins. Presently, up to 45 parameters can be simultaneously and reliably quantified with the CyTOF technique. In theory, CyTOF is capable of isotropic discrimination of more than 100 elemental masses, which suggests even higher multiplex capability in future applications. This enables CyTOF profiling and screening in a comprehensive and system‐wide manner. Second, given the precise isotopic discrimination of mass spectrometer, the channels in TOF have little cross talk and the need for compensation is limited,^3^ which to a great extent circumvents the time‐consuming and laborious compensation issues in conventional fluorescence flow cytometry. Next, the detection of these metal isotopes, which are absent or extremely rare in biological systems, would fundamentally resolve autofluorescence issues and background noises problems. In addition, with unique palladium‐based barcode labels,^4^ palladium‐tagged β2‐microglobulin‐based barcode label combinations,^5^ or ratiometry‐based CD45 barcode labels,^6^ individual samples from a large cohort can be pooled together for further analysis to eliminate batch effect. The commercial palladium‐based barcode labels adopt a 6‐choose‐3 barcoding scheme and allow 20 samples barcoded together, whereas the palladium‐tagged β2‐microglobulin‐based barcode label combinations adopt a 5‐choose‐2 scheme and enable 10 samples together. It may take multiple barcode sets in large‐scale experiments. The ratiometry‐based CD45 barcode labels based on three metals and three ratios can barcode 19 samples and as the ratio levels and mass tags numbers increase, the maximum potential barcoding capability would expand exponentially. This would make barcoding and consistent staining for large‐cohort study feasible, but at the expense of detection channel numbers. The rate of cell acquiring and analysis of CyTOF instrument is ∼500 cells/s, which permits the analysis of millions of cells in 1 h. More importantly, CyTOF enables in‐depth analysis, including exploration of signaling pathway alterations on archival samples, such as curated formalin‐fixed paraffin‐embedded tissues (FFPEs).^7^ With the advent of imaging mass cytometry (IMC), even spatial information and cell interactions in curated FFPEs can be obtained.^8^ Last, coupled with advanced computational tools and well‐established pipelines for high‐dimensional data analysis, CyTOF facilitates the visualization of immunocytes and their networks.

**TABLE 1:** Comparisons between flow cytometry and CyTOF properties

	Flow cytometry	CyTOF
Labeling	Fluorochrome	Heavy metal
Detector	Fluorescence detector	Mass spectrometry
Multiplex	Up to 30	Up to 45
Sensitivity	High	Low
Sample efficiency	>95%	<50%
Accessibility	Easy	Moderate
Cell throughput	10 000 cells/s	500 cells/s
Cost	Moderate	High
Sorting	Yes	No
Cell recovery	Yes	No
Data analysis	Simple and user‐guided	Complex bioinformatics

A large body of work has been exerted on the establishment and recapitulation of conventional fluorescence flow cytometry assays. For instance, the carrier strategy that significantly reduces required sample amount in fluorescence cytometry has been successfully adapted in CyTOF technique to enable the analysis of rare and precious clinical samples.^9^ Limitations and constraints of the CyTOF technique, however, still exist, as compared with flow cytometry (Table 1). First, cells cannot be recovered for further functional analysis using CyTOF technique, as cells are fixed and ultimately ionized. Second, the sensitivity of CyTOF is 10‐fold less than that of flow cytometry, limited by the chelating polymer used in CyTOF to attach metal reporter ions.^2^ Further, a sizable portion of cells is lost during the sample treatment and instrument processing, resulting in less than 50% of cells available for analyses by CyTOF. Flow cytometry, however, can measure over 95% of cells in a sample.^10^ In addition, as mass cytometry is mostly dependent on antibodies, careful antibody panel design and validation are required to ensure accurate and specific detection of all targets. Experienced and extensive labor are needed, especially when processing larger panels. Last, both the instrument and the reagents are expensive. The low accessibility of CyTOF instrument and high expense of the assay currently limit its wide utilization.

## 4 FUNCTIONAL ASSAYS

Ongoing improvements to CyTOF approaches continue to open new opportunities for implementing various functional assays to tackle the complexities of cellular immunology. Here, we provide an overview of CyTOF‐based functional assays.

### 4.1 Phenotype characterization

**FIGURE 2:**
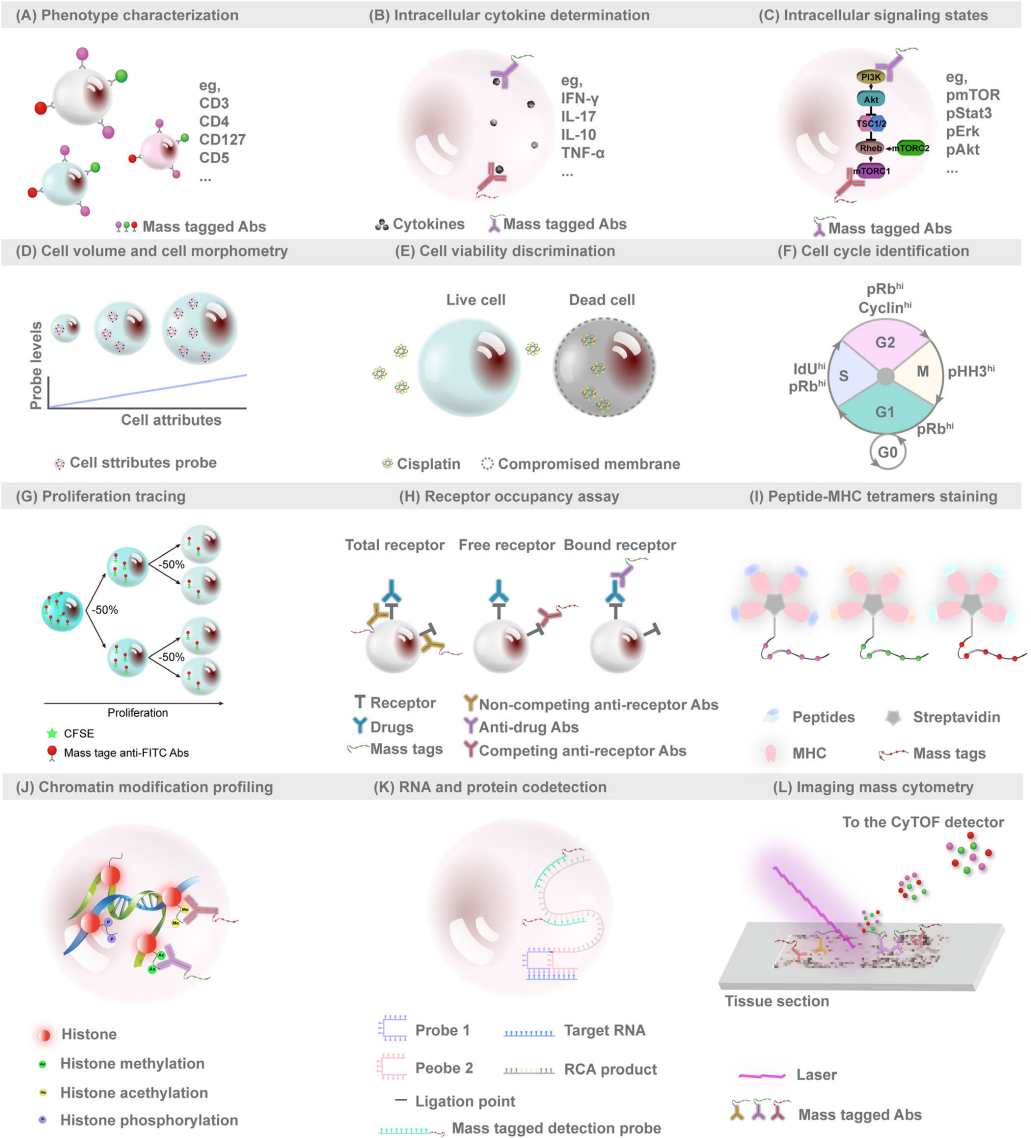
Schematic illustrations of functional assays using CyTOF. The current innovations in functional assays can be separated into 12 categories: **(A)** phenotypic characterization; **(B)** intracellular cytokines determination; **(C)** intracellular signaling state characterization; **(D)** cell volume and size measurement; **(E)** cell viability discrimination; **(F)** cell cycle identification; **(G)** proliferation tracing; **(H)** receptor occupancy assay; **(I)** tetramers‐based antigen‐specific T‐cell screening; **(J)** chromatin modification profiling; **(K)** RNA and protein codetection; and **(L)** imaging mass cytometry

CyTOF can multiplex up to 45 cellular markers with limited need for spectral overlap compensation, opening up a post‐fluorescence era of cytometry well suited for deep phenotyping of cells in complex systems. Genetically similar or even identical cells that play distinct roles in disease pathogenesis could be distinguished with CyTOF, hosting important implications for personalized medicine. Being able to quantitatively probe nearly any feature (Figure 2A), CyTOF can characterize various cell types^11^ including rare cells across the immune cell continuum.^12^

### 4.2 Intracellular cytokines determination

Intracellular cytokine profiling offers novel perspectives on immune activation states. The higher multiparameter capacity in CyTOF renders it an attractive instrument in intracellular cytokine staining assays (Figure 2B). Vendrame et al utilized CyTOF to evaluate the effects of cytokines on the natural killer (NK) cells and revealed that interleukin (IL)‐12/IL‐15/IL‐18 stimulation induced dramatically increased interferon‐γ (IFN‐γ) expression in NK cells.^13^ Doyle et al characterized plasmacytoid dendritic cells (pDCs) in the liver and peripheral blood of patients with hepatitis C virus (HCV) infection and demonstrated that liver pDCs were polyfunctional and capable of producing abundant IFN‐γ and other immune modulators during chronic HCV infection.^14^ With ever‐growing reports utilizing CyTOF to determine intracellular cytokines, we believe that CyTOF will likely act as an indispensable tool in immune cell function studies.

### 4.3 Intracellular signaling state

Cellular circuits sense environmental stimuli and accordingly attune the signaling network to enable key decisions regarding cellular response. Using metal‐chelated antibodies targeting phosphorylated proteins, CyTOF enables interrogation of signal propagation within individual cells. Studies have demonstrated the utility of CyTOF to assess cell signaling states in kinetic or time point (Figure 2C).^15^, ^16^ Shinko et al have provided an optimized protocol of phosphorylated signaling proteins staining for clinical blood samples.^17^ Combined with lineage markers and stimulations or inhibitions, the signaling states and cellular responsiveness are comprehensively evaluated.^16^, ^18^, ^19^

### 4.4 Cell volume and size measurement

Cell size and volume are fundamental characteristics that impact the structure and functions of any given cell type. Fluorescence flow cytometry uses the metrics of light scatter properties (forward and side scatter intensity) to determine cell volume and size. Stern et al established wheat germ agglutinin‐based and Osmium tetroxide‐based plasma membrane staining to gauge the size of mammalian cells.^20^ Rapsomaniki et al identified the ruthenium complex ASCQ_Ru as robust marker for quantifying cell volume.^21^ These stains (Figure 2D) substitute for light scatter properties evaluated in fluorescence flow cytometry and expand the range of parameters measured via CyTOF.

### 4.5 Cell viability discrimination

Discriminating cellular viability is critical in biological sample analysis, especially in functional studies such as intracellular signaling or drug responses, as it enables the removal of nonviable cells. Platinum‐based covalent viability regent,^22^ Cisplatin, was developed to label cells for CyTOF discrimination of live/dead ratios, on the basis that Cisplatin preferentially labels nonviable cells (Figure 2E).

### 4.6 Cell cycle identification

Cell cycle alterations are important aspects in tumor progression, developmental biology, and immune modulation. Behbehani et al developed a novel CyTOF approach to delineate cell cycle stages, based on iododeoxyuridine to mark cells in the S phase, together with antibodies against phosphorylated retinoblastoma, cyclin B1, cyclin A, and phosphorylated histone H3 to define G0, G1, G2, and M phases (Figure 2F).^23^ Protocols of cell cycle identification were well developed and detailed.^24^ Utilizing this cell cycle identification method, researches have revealed cell cycle differences that mediated chemotherapy sensitivities of acute myeloid leukemia^25^ and erythropoiesis impairment in telomerase knockout mice.^26^

### 4.7 Proliferation tracing

Good et al provided a dye dilution protocol for cell proliferation tracing across time and states, which could be widely applied in directing studies of cellular differentiation.^27^ A dilution assay of carboxyfluorescein succinimidyl ester (CFSE) was adapted and metal‐labeled anti‐fluorescein isothiocyanate antibody was employed to track CFSE signal changes (Figure 2G). Dividing cells that pass half CFSE signals to individual offspring cell act as a proxy for cell division counting. The proliferation tracing method helps uncouple the phenotypic and functional transitions in tandem with the sequence of cellular differentiation. Good et al used 23 markers and the proliferation tracing approach to track single naïve human T cells. A map of cell variations during naïve T cell expansion was built and revealed that undivided cells represent a large portion of phenotype diversity.

### 4.8 Receptor occupancy assay

The ratio of drug‐bound receptor to total receptor on individual cells is known as receptor occupancy. It represents a response biomarker for treatment of therapeutic monoclonal antibodies. Punet‐Ortiz et al^28^ employed a multiparametric quantitative flow cytometry to monitor CD49d receptor occupancy on peripheral blood mononuclear cells (PBMCs) of 19 multiple sclerosis patients receiving natalizumab therapy for a 6‐month follow up. They used this index of CD49d receptor occupancy to determine a safe, personalized regimen, and proposed an optimized CyTOF‐based receptor occupancy measurement.^29^ CyTOF enables the measurement of receptor occupancy in conjunction with more markers within wide varieties of cell types in a more reliable and reproducible manner. It would be valuable in drug pharmacodynamics or immune suppression fields, replacing fluorescence flow cytometry. Huse et al emphasized that receptor occupancy determination by CyTOF expands the clinical cytometry toolbox and introduced three basic formats of CyTOF‐based receptor occupancy assays (Figure 2H).^30^

### 4.9 Tetramers‐based antigen‐specific T‐cell screening

To better develop vaccines and targeted therapies for autoimmune diseases and cancer, cognate‐specific antigens and binding affinity of antigen‐binding T cells need to be comprehensively characterized. Studies have emphasized the adaptability of mass cytometry to profile T‐cell responses that are antigen specific and screen T‐cell reactivity against various major histocompatibility complex (MHC)‐class‐restricted epitopes. A modified peptide‐MHC class I tetramer was conjugated to metal chelating polymers, allowing multidimensional analyses by CyTOF (Figure 2I).^31^ Coupled with a combinatorial staining approach, Newell et al simultaneously tested hundreds of neoantigens in cancer.^32^, ^33^, ^34^ Antigen‐specific CD8^+^ T cells were screened with highly multiplexed combinatorial tetramer staining in a cohort of 14 nonsmall cell lung carcinoma patients before and after atezolizumab treatment,^33^ including eight responder patients and six with progressive disease status. The results indicated enrichment of low‐differentiated effector neoantigen‐specific CD8^+^ T cells in responders to atezolizumab treatment. Although lower affinity between T‐cell receptor and peptide‐MHC II and lower frequencies of antigen‐specific CD4^+^ T cells render peptide‐MHC II tetramers screening by CyTOF challenging, peptide‐MHC II tetramer staining in PBMCs and tissue‐derived cell suspensions was achieved after optimization.^35^, ^36^

### 4.10 Chromatin modification profiling

Histone modifications are fundamental to proteomic epigenetic regulation. Recently published studies employed CyTOF to investigate chromatin modification.^37^, ^38^ Cheung et al proposed epigenetic landscape profiling using CyTOF (EpiTOF), a highly multiplexed form to analyze histone modifications and identify dysregulations associated with immune‐mediated diseases at the single‐cell resolution (Figure 2J).^38^ Strategies of chromatin marks manipulation were established, such as ectopic overexpression or CRISPR‐ or RNAi‐mediated chromatin‐modifying enzymes depletion, to validate and select antibodies for EpiTOF. Two antibodies that are able to recognize total histone proteins were integrated into EpiTOF for variation control of antibody background, nuclear epitope accessibility, and histone expression. The investigators applied EpiTOF to examine 24 healthy cytomegalovirus‐seronegative subjects, focusing on 22 major immune subsets and examined the cellular levels of four histone variants in eight classes of histone modifications. The results indicated that chromatin variations increased with age, which are largely driven by nonheritable factors, and cell‐type‐specific chromatin mask profiles predicted identity of immune cells.

### 4.11 RNA and protein codetection

To enable the codetection of RNA and protein signatures at the single‐cell resolution, Frei et al developed the proximity ligation assay for RNA (PLAYR) method, which is a proximity ligation assay by CyTOF for highly multiplexed transcript quantification (Figure 2K).^39^ PLAYR adopts pairs of DNA oligonucleotide probes that include a region to hybridize target transcripts and another region as a template to bind and circularize two additional oligonucleotides, which are ligated and amplified through rolling circle amplification. The amplified products of each probe pair are detected using oligonucleotides labeled with mass tags. Another novel CyTOF‐based mRNA transcript and protein codetection method, termed metal in situ hybridization (MISH), was developed via combination of CyTOF and RNAscope@ platform.^40^ The technique enables signal amplification through hybridizing RNA‐specific probes and binding with amplified sequence‐targeting metal‐labeled probes. Both PLAYR and MISH are compatible with routine immunostaining, and simultaneous transcripts quantification number is only limited by the number of reporters that can be conjugated to the oligonucleotides. Notably, in both methods, the measurement of RNAs will occupy the mass channels, which means the total number of simultaneously codetected RNAs and protein targets depends on the number of mass channels (up to 45 at present). Frei et al^39^ gave an example of simultaneous multiplexed profiling of protein and transcripts. They used PLAYR to monitor the induction of eight cytokine transcripts and 18 protein epitopes in PBMCs after stimulation with lipopolysaccharide and revealed correlations between the functional capacity of each cell and its protein marker expression.

### 4.12 Imaging mass cytometry

Geisen et al used CyTOF to image tissue samples to acquire spatial proteomics.^8^ The proposed technique, IMC, uses laser ablation with a resolution of 1 μm to generate plumes of tissue sections that are aerosolized, atomized, ionized, and carried to the mass spectrometry detector by an inert gas stream (Figure 2L). IMC is considered as a landmark development, as it allows for simultaneous profiling of up to 50 parameters in tandem with cell interaction and spatial information at subcellular resolution. Since its introduction, IMC is being rapidly adopted for various applications.^41^, ^42^, ^43^, ^44^, ^45^, ^46^, ^47^ Damond et al employed IMC to investigate 1581 islets from four nondiabetic, four patients of onset type 1 diabetes, and four patients of long‐duration type 1 diabetes.^44^ They depicted the progression of human type 1 diabetes and revealed an alteration of β cells phenotype prior to its destruction.

## 5 CyTOF DATA ANALYSIS

Prior to analysis, a stringent beads‐based data normalization is required^48^ to correct variations in instrument performance caused by drift and build‐up of cellular debris. For samples run individually or in multiple barcode sets, a batch adjustment technique is required. Schuyler et al^49^ flexibly adopted several standard normalization methods, such as per channel quantile normalization, and recommended combining statistical testing results of conditions within batches via Fisher's method. Shaham et al^50^ presented a deep learning approach, based on distribution‐matching residual networks, to effectively attenuate batch effects. In both methods, technical replicates, as anchors or references, are included in each run, allowing direct estimation and adjustment of batch effect. CyTOF achieves analysis of high cell throughput (up to millions of cells) and high dimensions, which pose challenges toward its analysis. Traditional user‐guided manual gating on bivariate plots has been proven as subjective, cumbersome, and inefficient. Novel computational analyses that are able to visualize and explain CyTOF‐generated data have been developed (Table 2). Each of these algorithmic tools is designed with distinct goals and advantages.

**TABLE 2:** Comparisons between flow cytometry and mass cytometry methods

Methods	Classification	Description	Ref.
PCA	Dimensionality reduction	Linear, principle component analysis, orthogonal transformation.	^51^
Isomap		Nonlinear, spectral clustering, geodesic distance.	^53^
Diffusion Map		Nonlinear, spectral clustering, diffusion distance.	^54^
t‐SNE/viSNE		Nonlinear, t‐distributed stochastic neighborhood embedding, attraction/repulsion balance.	^55^
UMAP		Nonlinear, uniform manifold approximation and projection, identify user‐specified number of neighbors to build high‐dimensional manifolds.	^56^
ACCENSE	Unsupervised clustering	t‐SNE, kernel‐based density estimation, peak‐finding, and partitioning.	^57^
Phenograph		k‐nearest neighbors (k‐NN) detection, community detection, and Jaccard similarity coefficient.	^58^
Xshift		Weighted k‐NN density estimation and density‐ascending path‐based clustering.	^59^
FlowSOM		Self‐organizing map, minimal spanning tree‐based nodes connection, and consensus of hierarchical meta‐clustering.	^60^
DEPECHE		Penalized k‐means clustering.	^61^
SPADE		Density‐normalization, spanning tree progression analysis, and hierarchical/agglomerative clustering.	^62^
ACDC	Semi‐supervised clustering	Community detection of landmark points. Cells and random walker‐based clustering.	^63^
LDA		Linear discriminant analysis.	^64^
Citrus	Clustering with statistics	Hierarchically clustering, regularized supervised learning algorithms, nearest shrunken centroid methods, and lasso regularized logistic regression.	^67^
Wanderlust	Differentiation trajectory determination	Ensemble of k I‐nearest neighbor graphs, shortest path distance‐based trajectory construction, and waypoints‐based iteratively trajectory refinement.	^69^

Of these tools, dimensionality reduction is utilized to organize complex data into recognizable patterns and thus provides an overview of the data. Principal component analysis (PCA)^51^ is a commonly employed dimensionality reduction technique for visualizing the multidimensional data.^52^ With linear transformation, PCA can reduce dimensionality; however, biological systems can contain nonlinear relationships. Therefore, nonlinear dimensionality reduction techniques that avoid representation of overcrowding have gradually gained attraction. Nonlinear dimensionality reduction techniques employed in CyTOF data analysis include Isomap,^53^ Diffusion Map,^54^ t‐distributed stochastic neighborhood embedding (t‐SNE/viSNE),^55^ and uniform manifold approximation and projection (UMAP).^56^ Of these, t‐SNE can efficiently present the local data structure and is commonly used in single‐cell data analysis. But the limitations of t‐SNE include global information loss, long computational time, and inability to provide meaningful results. Compared to t‐SNE, UMAP can preserve more data structure during a shorter run time.

Other computational tools further define cell clusters. Clustering approaches can be divided into unsupervised^57^, ^58^, ^59^, ^60^, ^61^, ^62^ and supervised^63^, ^64^ categories. Unsupervised clustering methods detect cell clusters mainly based on protein expression profiles from a single, multiple, or combined biological sample. Unsupervised clustering detects cell subsets in a data‐driven and unbiased manner that facilitates exploratory analysis of previously unknown cell subpopulations. Defined clusters can be individually analyzed or compared across samples in different biological conditions. For example, ACCENSE combines t‐SNE mapping with discrete cell clusters identification.^57^ With a peak‐detection algorithm that identifies local maxima, ACCENSE is able to partition a two‐dimensional t‐SNE map. Clusters are decided by a user‐specified *P*‐value threshold. Corresponding expression profiles are generated with built‐in functions for each detected cluster. ACCENSE produces color‐coded and density‐partitioned cluster maps, facilitating visualization and comparison. Supervised clustering methods usually depend on biological or clinical variables that describe each sample, such as disease status or clinical outcome. The external information can be used to train an interpretable prediction model. For instance, defined clusters that correlate with clinical outcomes can be considered as biomarkers. Automated cell‐type discovery and classification (ACDC)^63^ and linear discriminant analysis (LDA) are typical supervised clustering methods. ACDC utilizes biological knowledge of a marker × cell type annotation table to guide learning algorithms. Trained with predetermined manual labels, LDA classifier^64^ achieves high clustering precision in automatically identification of cell populations. Weber et al^65^ and Liu et al^66^ provided detailed comparison frameworks and guidelines of clustering methods.

Furthermore, some computational tools provide statistical testing or infer differentiation trajectories. Citrus^67^ (cluster identification, characterization, and regression) aims to identify cell abundance or cellular traits associated with disease conditions used to categorize samples. Conditions generally include healthy control or patients, before or after/with or without therapeutic intervention, cancerous or paracancerous tissue, responsive or irresponsive to treatment, and good or poor prognosis. In addition to hierarchically clustering of phenotypically similar cells, Citrus employs regularized supervised learning algorithms including L1‐Penalized Regression (glmnet) and Prediction Analysis for Microarrays to identify predictive cluster features. Gaudillière et al applied Citrus and identified a signature that correlated with patient recovery after hip surgery.^68^ Wanderlust sequentially orders cells based on their developmental trajectory and enables inference of the differentiation course. First, the data are transformed into an ensemble of graphs, then waypoints are randomly selected. Next, an orientation trajectory is calculated based on a user‐defined “initiate” cell, which is further refined by waypoint cells. Wanderlust produces a trajectory over an average of all graphs. Within the results, each marker can be plotted against the trajectory axis to further examine trends. Wanderlust was used to build and identify novel transitional phenotypes in B‐cell development by extending a trajectory map from hematopoietic stem cells to naïve B cells.^69^

Novel tools that are faster and containing more features are being continuously developed and proposed. In addition, proper combination of computational approaches may provide more insights into the interpretation of the true complexity of biological systems. Therefore, comprehensive understanding of the functionality, the strengths, as well as the limitations of available algorithms is critical to the selection of optimal analytical tool for specific goals.

## 6 APPLICATIONS

The benefits of CyTOF are obvious in the immunology field. In combination with novel bioinformatics techniques, CyTOF allows for the systematic investigations of fundamental questions on immune pathogens and immune mechanisms underlying clinical disease manifestations. A wide range of applications for either mice or humans including studies of cancer, immunotherapy, autoimmune diseases, infective diseases, cardiovascular disease, and neuron science are described below.

### 6.1 Cancer

With relative high cellular throughput and high dimensions, CyTOF is an ideal and potent technique that reveals intratumor heterogeneity in a variety of tumors, including breast,^70^, ^71^, ^72^, ^73^ lung,^74^, ^75^, ^76^, ^77^ oropharyngeal,^78^ brain,^79^, ^80^, ^81^, ^82^, ^83^ colon,^7^, ^11^, ^84^, ^85^ ovarian,^86^, ^87^ kidney,^88^, ^89^ gastric cancer,^90^, ^91^ leukemia and lymphoma,^19^, ^92^, ^93^, ^94^, ^95^ and melanoma.^96^, ^97^ A summary of single‐cell CyTOF studies on primary tumors of various human cancers is presented in Table 3. Beyond the analysis of phenotypic markers,^88^ CyTOF allows simultaneous measurement of cell signaling process^7^, ^85^, ^92^, ^93^ through the analysis of protein phosphorylation and neoantigen‐specific T‐cell pools. In addition to peripheral blood profiling,^81^ single‐cell suspensions dissociated from organs or tumor tissues^75^, ^79^, ^82^, ^88^ can also disentangle alterations in the local immune networks.

**TABLE 3:** Summary of CyTOF studies on various types of human cancers

Organs	Tumor types	Cell source	Multiplex	Results	Reference
Breast	Breast cancer	Tumor tissues	35‐plex/38‐plex	Advanced estrogen receptor (ER)^+/−^ tumors exhibited higher percentages of programmed death ligand 1 (PD‐L1)^+^ macrophages and exhausted T cells.	^70^
	4T1 metastatic breast cancer	PBMC/spleen	24‐plex	Cisplatin downregulated splenic CD44^+^interleukin (IL)‐17A^+^ myeloid‐derived suppressor cells (MDSCs) and promoted circulating interferon (IFN)‐γ^+^ myeloid cells.	^71^
	Breast carcinoma	Tumor tissues	38‐plex	Intratumor T‐cell subsets exhibited diverse patterns of environmental signatures.	^72^
	Invasive breast lobular carcinoma in mouse model	Mammary glands	33‐plex	Immune suppression and exhaustion were observed in myeloid and T‐cell compartments in mice bearing cancer.	^73^
Lung	Early stage lung adenocarcinoma.	PBMC/tumor tissues	31‐plex	Lung macrophages prevalent in noninvolved lung tissue contained higher levels of cerium, whereas it was lower in tumor‐associated macrophages.	^74^
	Nonsmall cell lung cancer	Tumor tissues	35‐plex	T‐cell immunoglobulin and mucin‐domain containing 3 (TIM‐3), lymphocyte‐activation gene 3 (LAG‐3), and programmed cell death 1 (PD‐1) showed differential functional impact, tissue/cell distribution, and clinical significance in nonsmall cell lung cancer.	^75^
	Nonsmall cell lung cancer	Tumor tissues	31‐plex	Developed EMT‐MET PHENOSTAMP for mapping epithelial‐mesenchymal transition (EMT) states.	^76^
	Early lung adenocarcinoma	PBMC/Tumor tissues	32‐plex/32‐plex/38‐plex	Early stage lung cancer exhibited increased peroxisome proliferators‐activated receptor γ (PPARγ)^hi^ macrophages, decreased CD141^+^ dendritic cells (DCs), and reduced and impaired NK cells.	^77^
Oropharynx	Oropharyngeal cancer	Tumor tissues	36‐plex	Intratumor human papillomavirus type 16 (HPV16)‐specific type I T cells and its oriented tumor microenvironment were present and related to a better overall survival.	^78^
Brain	Glioblastoma/ multi‐tumor	PBMC	36‐plex	CD73 was identified as a combinatorial immunotherapeutic target.	^79^
	Glioblastoma	Tumor tissues	28‐plex	An increase in cytotoxic immune infiltration.	^80^
	Glioblastoma	PBMC	25‐plex	MDSC reduction was associated with a continued increase of dendritic cells (DCs).	^81^
	Gliomas/brain metastases	Tumor tissues	37‐plex/36‐plex	Brain metastases showed upregulated invasion of T cells and monocyte‐derived macrophages and gliomas characterized by activated microglia.	^82^
	Glioma	PBMC/Tumor tissues	27‐plex	T cells that expressed PD‐1 displayed hallmarks of activation and exhaustion.	^83^
Colon	Colorectal cancer	FFPE	20‐plex	Dysregulation of signaling pathways in colorectal cancer.	^7^
	Microsatellite stable colorectal cancer	PBMC/tumor tissues	27‐plex	Increased immunosuppressive/exhausted T cells at tumor lesions.	^11^
	Colon cancer	PBMC	19‐plex	Abnormal levels of epithelial cell adhesion molecule (EpCAM)^+^CD4^+^ T cells were observed in colon cancer patients.	^84^
	Advanced colorectal cancer following chemotherapy	PBMC	34‐plex	Sustained reduction in CD16^+^ natural killer cells (NKs) following chemotherapy in colorectal cancer patients.	^85^
Ovary	Ovarian cancer	PBMC/Tumor tissues	36‐plex	Tregs with highly activated phenotype were present in ovarian cancer,	^86^
	High grade serous ovarian cancer	Tumor tissues	41‐plex	Higher frequencies of cMyc^+^HE4^+^vimentin^+^ cell subset were observed in tumors from patients with poorer outcome.	^87^
Kidney	Clear cell renal cell carcinoma	Tumor tissue	35‐plex/33‐plex	Immune compositions correlated with progression‐free survival.	^88^
	Different renal tumors	Tumor tissues	28‐plex/21‐plex	Different renal tumors had different cell subsets with distinct characteristics.	^89^
Stomach	Gastric cancer	Tumor tissues	32‐plex	CD8^+^ T and FOXP3^+^CD4^+^ T cells were important markers for diagnosis of gastric cancer.	^90^
	Gastric cancer	AGS cells	17‐plex	CyTOF technology was critical at single‐cell analysis of gastric cancer.	^91^
Blood	Myelofibrosis/secondary acute myeloid leukemia	PBMC/BM	35‐plex	NF‐kB signaling was abnormally activated.	^19^
	Secondary acute myeloid leukemia	PBMC	29‐plex	Patients with thrombopoietin stimulation exhibited higher levels of signal transducers and activators of transcription (STAT) phosphorylation in Lin^−^CD61^+^CD34^−^CD38^−^CD45^low^ cells.	^92^
	B‐cell precursor acute lymphoblastic leukemia	BM	35‐plex	Pre‐B‐cell receptor signaling‐activated pre‐BI cell and mTOR signaling‐activated pro‐BII cells are related with relapse.	^93^
	Germinal center B‐cell lymphoma	Tumor tissues	32‐plex	In addition to CD68 and CD163, S100A9, CCR2, CD32, CD36, and Slan were also critical in the characterization of lymphoma‐specific tumor macrophages.	^94^
	Follicular lymphoma	Tumor tissues	33‐plex	Patient survival was correlated with naïve CD4^+^ T‐cell frequency and CD27^−^CD28^−^ T cells frequency.	^95^
Skin	Stage IV melanoma	PBMC	38‐plex	The alterations in myeloid phenotypes and differentiated NKs were associated with patient survival.	^96^
	B16 melanoma	Tumor tissues	Unknown	General control nonderepressible 2 (GCN2) altered function of macrophages and MDSCs in tumor microenvironment of melanoma.	^97^

Abbreviations: BM, bone marrow; FFPE, formalin‐fixed paraffin‐embedded sections; PBMC, peripheral blood mononuclear cells.

It is evident that CyTOF can potentially identify valuable phenotypic and functional variations, including cytokines and phosphor‐signaling alterations, in the course of tumor development, progression, and metastasis. This enables researchers to identify defined populations for deep causative mechanism studies. Moreover, CyTOF permits biomarker discovery for disease diagnosis or prognosis prediction, because multiple parameters can be measured at the single‐cell resolution from clinical samples of different conditions. For example, Good et al utilized CyTOF to simultaneously quantify 35 phenotypic and signaling‐associated proteins in the B‐cell development of 60 primary diagnostic patients with B‐cell precursor acute lymphoblastic leukemia.^93^ They employed machine learning to analyze the high‐dimensional data and identified activated and responsive pre‐B‐cell receptor signaling in pre‐BI cells, and activated mTOR signaling in pro‐BII sunsets was sufficient to predict patient relapse at diagnosis. By following a data‐driven approach, this study provides a framework for applying CyTOF in human cancer diagnosis and prognosis prediction.

### 6.2 Immunotherapy

The heterogeneous and suboptimal clinical responses to immunotherapy treatment highlight the need for deep profiling of systemic immune networks to support studies of pathogenesis, disease tracking, therapeutic targets identification, and treatment selection. Spitzer et al^98^ demonstrated the utility of CyTOF to profile systemic immune orchestrations with multiparameters in peripheral blood and tissue, including tumor, lymph node, spleen, and bone marrow. They studied three groups of mice with spontaneous model of triple‐negative breast cancer: untreated, effective, and ineffective immunotherapy. The investigators sought to define immune system alterations in the tumor environments between effective and ineffective treatment group. Network analysis identified that CD4^+^ T cells initiated immunotherapy response and conferred protection against new tumors. Researches further confirmed the functional differences of T‐cell compartments in response to immunotherapy, both in peripheral blood^99^ and in tumor infiltrates.^100^ Although current immune checkpoint therapies target mainly lymphoid compartments, researches have started to focus on the role of myeloid compartments in response of immunotherapy (Table 4).^101^, ^102^, ^103^ In addition, in combination with immunotherapy and by elevating the efficacy and duration of immune responses, CyTOF offers an attractive promise in developing more effective treatment schemes. Beyrend et al used CyTOF to decipher the rational design of combination immunotherapy and concluded that PD‐L1 blockade therapy was enhanced by therapeutically co‐targeting activating and inhibitory (LAG3/PD‐1) molecules.^104^ Chua et al focused on the synergy between radiotherapy and immune checkpoint blockade and found that expansion of activated Ki‐67^+^CD8^+^ T cells may account for its synergism relationship.^105^ Although challenging, CyTOF profiling should offer novel insights into personalized immunotherapy.

**TABLE 4:** Summary of CyTOF studies in the fields of immunotherapy, autoimmune disease, infective diseases, cardiovascular diseases, transplantation, and neuroscience

Fields	Specific fields	Cell source	Multiplex	Results	Reference
Immunotherapy	Triple‐negative breast cancer	PBMC/tumor infiltrates/BM/lymph node/spleen	41‐plex	Effective cancer immunotherapy is dependent on systemic immunity.	^98^
	Stage IV Melanoma	PBMC	28‐plex	Clinical response is correlated with the ratio of T‐cell reinvigoration to tumor burden.	^99^
	MC38 colorectal tumors	Tumor infiltrates	29‐plex	Distinct cellular mechanisms were utilized by anti‐programmed cell death 1 (PD‐1) and anti‐cytotoxic T‐lymphocte associated protein 4 (CTLA‐4).	^100^
	Stage IV melanoma	PBMC	30‐plex/ 26‐plex/ 25‐plex	CD14^+^CD16^−^HLA‐DR^hi^ monocytes frequency predicts anti‐PD‐1 immunotherapy response.	^101^
	T3 sarcoma	Tumor infiltrates	37‐plex	Immune‐checkpoint therapy was critical in macrophages polarizing in the milieu.	^102^
	Melanoma	PBMC	36‐plex	Identified distinct biomarkers for anti‐CTLA‐4 and anti‐PD‐1 therapy.	^103^
	MC38 colorectal tumors	Tumor infiltrates	38‐plex	Programmed death ligand 1 (PD‐L1) blockade upregulated specific tumor‐infiltrating CD4^+^ and CD8^+^ T‐cell subsets.	^104^
	Metastatic disease of various tumor histology	PBMC	40‐plex	Upregulated Ki‐67^+^CD8^+^ T cells may be correlated with the synergy between radiotherapy and Immunotherapy.	^105^
Autoimmune disease	Rheumatoid arthritis (RA)	Joint tissue	36‐plex	The synovium of patients exhibited expanded PD‐1^hi^CXCR5^−^CD4^+^ T cells.	^106^
	RA	PBMC	32‐plex	Patients exhibited upregulated CD27^−^HLA‐DR^+^ effector memory cells.	^107^
	RA	synovial tissue	34‐plex	Expanded cells associated with rheumatoid arthritis synovia.	^108^
	RA	PBMC	33‐plex	RA induces the expansion of CD11b^low^ neutrophils.	^109^
	Systemic sclerosis, systemic lupus erythematosus (SLE), and primary Sjögrens syndrome	PBMC	34‐plex	All autoimmune diseases exhibited varied frequencies of immune‐cell subsets, with low discriminative power.	^110^
	Juvenile idiopathic arthritis	PBMC	37‐plex	Relapse patients had CD3^+^CD4^+^CD45RA^−^tumor necrosis factor α (TNFα)^+^ PD‐1^−^CD152^−^ T cells prior to therapy withdrawal.	^111^
	SLE	PBMC	40‐plex	Toll‐like receptors (TLR)‐induced responses within cell types diverse.	^112^
	SLE	PBMC	33‐plex	Patients taking mycophenolate mofetil had significantly decreased transitional B cells, plasmablasts, and T cells.	^113^
	Psoriasis	PBMC	31‐plex	Psoriasis was impacted by CD3^–^CD4^+^ cells.	^114^
	Atopic dermatitis and psoriasis	PBMC	42‐plex	Mucosal‐associated invariant T cells, recirculating memory CD8^+^, and CD49^+^CD4^+^ T cells play a role in atopic dermatitis.	^115^
	Early multiple sclerosis (MS)	PBMC	64‐plex	Early MS‐PBMCs exhibited upregulated CCR7^+^ and interleukin (IL)‐6^+^ T cells, whereas NFAT1^hi^T‐bet^hi^CD4^+^ T and CD141^hi^IRF8^hi^CXCR3^+^CD68^−^ dendritic cells decreased.	^116^
	Neuroinflammation and neurodegeneration	PBMC and brain infiltrates	39‐plex	Myeloid cells are distinct in different mouse neuroinflammation and neurodegeneration model.	^117^
	Relapsing‐remitting multiple sclerosis	PBMC	35‐plex	T helper cells expressing granulocyte‐macrophage colony‐stimulating factor and the CXCR4 expanded in patients with multiple sclerosis.	^118^
Infectious diseases	Salmonella Typhi infection	PBMC	30‐plex	Adults and older pediatric patients had more multifunctional effector memory T (TEM) and effector memory CD45RA^+^ T clusters than children.	^119^
	Salmonella Typhi infection	PBMC	42‐plex /42‐plex	Salmonella infection induced accumulation of circulating interferon (IFN)‐γ‐ and macrophage inflammatory protein 1 β (Mip‐1β)‐ producing CD38^+^CCR7^−^CD4^+^ T cells.	^120^
	Mycobacterium tuberculosis infection	PBMC	38‐plex	Metformin intake induced decreased CD14^hi^CD16^−^ classical monocytes and increased CD14^−^CD16^+^ nonclassical monocytes	^121^
	Mycobacterium tuberculosis infection	PBMC	37‐plex /40‐plex	Enhanced cytotoxic responses and continuous inflammation is related to latent tuberculosis.	^122^
	Streptococcus pneumoniae	Nasal biopsy	37‐plex	Colonized clusters had significantly lower B cells and CD161^+^CD8^+^ T cells than noncolonized controls.	^123^
	Influenza A virus infection	PBMC	38‐plex	The 2009 pandemic H1N1 strain (Cal/09) versus a seasonal 2011 H3N2 strain (Vic/11) infection was predicted with CD54 and CD112 natural killer (NK) cell‐activating ligands.	^124^
	Human immunodeficiency virus (HIV) infection	Tonsil	38‐plex	HIV entry but not viral gene expression was supported by memory CD127^hi^CD4^+^ T cells in HIV patients.	^125^
	HIV infection	PBMC	26‐plex	Noticeable amounts of CD25^−^DR^−^CD4^+^ “resting” T cells were into cycle or expressed coinhibitory molecules.	^126^
	HIV infection	CD4^+^ T cells	19‐plex	IL‐15 stimulation induced expansion of memory and memory stem CD4^+^ T cells.	^127^
	HIV infection	lymph nodes	37‐plex	An oligoclonal HIV‐reactive IL‐21^+^ follicular helper T cells accumulated in severe HIV patients and correlated with abnormal B‐cell distribution.	^128^
	HIV infection	PBMC	28‐plex	CD27^hi^CD28^hi^CD127^hi^CD44^hi^CD4^+^ T cells were abundant in healthy subjects and acute stage patients undergoing antiretroviral therapy.	^129^
	HIV infection	PBMC	29‐plex	HLA‐I^+^CD64^+^LILRA2^+^ LILRB4^+^CD317^+^ monocytes were plentiful in early HIV‐infection and CD32b^+^HLA‐DR^+^CD1c^+^ classic dendritic cells (cDCs) were abundant in HIV controller patients.	^130^
	HIV infection	PBMC	35‐plex/ 32‐plex/ 33‐plex	Monocyte and PMNs displayed upregulations of CD11a, CD11b, CD32, CD38, CD64, CD83, CD86, and TLR2 in HIV‐infected patients.	^131^
	HIV infection	Bronchoalveolar lavage cells	7‐plex	Expression of CD163 significantly decreased in HIV‐infected subjects, and CD163 was inversely correlated with cytochrome P450 family 1 subfamily B member 1 (CYP1B1) expression in alveolar macrophages.	^132^
	HIV infection	PBMC	38‐plex	NKG2C and CD2 expression were increased; CD244 and NKp30 expression were decreased in IL‐2‐treated NK cell repertoire in treated HIV‐infected patients.	^133^
	Japanese Encephalitis virus infection	Brain	9‐plex	CD8^+^ T cells infiltration was presented in the central nervous system of mice after infection.	^134^
	Ebola virus infection	PBMC	42‐plex	Nonclassical monocytes and myeloid DCs were dramatically reduced in patients. Declining viral load correlated with increased classical monocyte and CD38‐upregulated plasmatoid DCs (pDCs).	^135^
	Primary gammaherpesvirus infection	Lung	35‐plex	Effector CD4 T cells were observed in the lungs of acutely infected mice, including an activated subset that co‐expressed IFN‐γ, TNF‐a, and IL‐10.	^136^
	Chikungunya virus infection	PBMC	37‐plex	Acute infection was associated with expansion of CD14^+^CD16^+^ monocytes.	^137^
	Hepatitis B virus (HBV) infection	PBMC	40‐plex	Serum HBsAg level variations did not correlate with phenotypes and functions of T and NK cells.	^138^
	HBV infection	PBMC	8‐plex	Circulating Vδ1^+^ and Vδ2^+^ γδT‐cells displayed distinct phenotypes and functions in patients with acute or chronic hepatitis B.	^139^
	Zika virus infection	PBMC	37‐plex	Acute patients exhibited elevated IFN‐β across major cell subsets.	^140^
	Dengue virus (DENV) infection	PBMC	37‐plex	Compared with the unstimulated cells, DENV IFN‐γ^+^ effector memory T cells had higher expression of activation and effector molecules.	^141^
	DENV infection	PBMC	32‐plex/29‐plex/40‐plex	Dengue infection caused broad activation in immune system and dengue‐specific T cells differentiated into two types.	^142^
	Zika virus infection	PBMC	37‐plex	Acute infection and convalescent stages exhibited differentially expanded CD14^+^ monocytes.	^143^
	Zika virus infection	Spleen	12‐plex	Significantly reduced inflammatory monocyte and neutrophil cellular responses were observed in the rectal route group.	^144^
	Corona virus disease (COVID‐19) pneumonia	PBMC	35‐plex	Immunosuppression and immune dysfunctions existed in COVID‐19 patients.	^145^
	COVID‐19 pneumonia	PBMC	Unknown	The IFN‐γ‐eosinophil pathway activated before lung hyper‐inflammation.	^146^
	COVID‐19 pneumonia	PBMC	35‐plex	Circulating CXCR3^+^CD4^+^ T, CXCR3^+^CD8^+^ T, and CXCR3^+^ NK cells were upregulated in severe patients and restored to normal levels after mesenchymal stem cell transplantation.	^147^
	Helminth infection	PBMC	37‐plex	Human type 2 and regulatory networks were heterogeneous in helminth‐infected patients.	^148^
	Malaria infection	PBMC	29‐plex	Approximately 80% of mature B cells that expanded after acute infection expressed CD11c.	^149^
Cardiovascular disease	Mouse atherosclerosis	Aorta	35‐plex	Aortic leukocyte system is as complex as that in lymphoid organs.	^150^
	Human atherosclerotic plaques	Plaque/PBMC	37‐plex	The atherosclerotic plaque are dominated by T cells and macrophages.	^151^
Transplantation	Kidney transplantation	PBMC	34‐plex	PD‐1^+^CD57^−^ exhausted T cells increased after lymphocyte‐depleting induction treatment, which correlated with better allograft function.	^154^
	Kidney transplantation	PBMC	33‐plex	Frequencies of transitional B cell and regulatory T cell at the baseline could discern between responders and nonresponders.	^155^
	Pediatric liver transplantation	PBMC	22‐plex	In operationally tolerant patients, the CD4^+^CD5^+^CD25^+^CD38^−/lo^CD45RA^−^ cells were upregulated in comparison with patients of low immunosuppression levels.	^156^
Neuroscience	Normal mouse brain	Brain/PBMC	44‐plex	CD44 discriminates infiltrating and resident myeloid cells in the brain.	^157^
	Postmortem human brain	Brain∖PBMC	57‐plex	Regional specific heterogeneity existed in human microglia.	^158^
	Homeostasis, epilepsy, or tumors	Brain	37‐plex/36‐plex	A unique glioma‐associated microglia was identified.	^159^
	Aging, Alzheimer's disease, and multiple sclerosis	Brain	43‐plex	Central nervous system border‐associated macrophages were distinguished by CD38 and major histocompatibility complex (MHC) II and all microglia are homogenously affected in neuroinflammatory disease.	^160^
	Neurodegeneration	Brain	33‐plex	Repopulated microglia showed IFN regulatory factor 7‐driven activation pattern.	^161^
	Alzheimer's disease	PBMC	21‐plex	Increased CD8^+^ TEM cells were observed in Alzheimer's disease.	^162^
	Refractory epilepsy and autoimmune encephalitis	PBMC	40‐plex	Patients with refractory epilepsy and autoimmune encephalitis displayed CD4^+^ and CD8^+^ T cell subsets alterations and unbalanced proinflammatory IL‐17 production. Refractory epilepsy patients uniquely showed NK cells alteration.	^163^
	Acute stroke	PBMC	38‐plex	Increased signal transducers and activators of transcription 3 (STAT3) signaling in innate immune cells in the acute phase, increased cAMP‐response element binding protein signaling in adaptive immune cells during the intermediate phase, and increased neutrophils and immunoglobulin M (IgM)^+^ B cells in the late phase were observed.	^164^

Abbreviations: BM, bone marrow; PBMC, peripheral blood mononuclear cells.

### 6.3 Autoimmune disease

Systemic activation of inflammatory cells plays a critical role in disease severity, progression, and therapy response of patients with autoimmune diseases. A deep understanding of heterogeneities in inflammatory states of individual patients during the disease course can contribute to therapeutic decisions. Many studies have used CyTOF to examine the pathogenesis of autoimmune diseases (Table 4).^18^, ^36^, ^106^, ^107^, ^108^, ^109^, ^110^, ^111^ Several researchers focused on the immunome perturbations in patients with rheumatoid arthritis, a chronic autoimmune, inflammatory disease depicted with synovitis in small‐ and medium‐sized joints,^18^, ^106^, ^107^, ^108^, ^109^ exploring both the peripheral blood^18^, ^107^ and synovium tissue samples.^106^, ^108^ In one study,^106^ the investigators examined synovitis of patients with rheumatoid arthritis through a 36‐plex CyTOF panel specific to activated T cells. The results identified upregulated PD‐1^hi^CXCR5^−^CD4^+^ T cells in patients, indicating a key functional role of CD4^+^ T subsets in rheumatoid arthritis. O'Gorman and coworkers focused on specific chemokine signature in Systemic lupus erythematosus (SLE) patients and identified Toll‐like receptor activation.^112^ Similarly, Slight‐Webb's groups applied CyTOF to reveal the STAT3 phosphorylation reduction after mycophenolate mofetil treatment in SLE.^113^ Another study, with the aid of CyTOF, interrogated the circulatory reservoir of CD4^+^ subsets in juvenile idiopathic arthritis patients undergoing TNF‐alpha therapy withdrawal and found putative subsets prior to withdrawal that discriminated relapse from remission.^111^ Researchers have also provided comprehensive overview of distinct immune signatures in multiple autoimmune conditions, including psoriasis,^114^, ^115^, ^116^ neuroinflammation,^117^ and multiple sclerosis.^118^ Together, these results provided a strong foundation for CyTOF studies with increased dimensionality to characterize central immune mediators in various autoimmune disorders.

### 6.4 Infectious diseases

Infection caused by a vast majority of microorganisms induces profound immune responses that involve innate and adaptive immune subsets. Recently, a plethora of studies applied CyTOF to investigate pathogen‐specific immunological signatures in infectious diseases (Table 4). Revealing bacteria's physiology and pathogenicity and the specific immune compartments that steer the immunological reactions to infection is crucial for vaccine development, diagnostic, and tailoring of treatment schemes. CyTOF has been gradually integrated into the research of bacteria‐associated diseases to identify pathogen‐specific immune signatures and characterize response disparity of leukocytes to vaccine. Rudolph et al studied T‐cell responses to HLA‐E‐restricted *Salmonella enterica* serovar Typhi antigen before and after Ty21a vaccination.^119^ Several multifunctional gut‐homing effector memory T and effector memory CD45RA^+^ T cells were more abundant in adult patients, compared with younger children. Napolitani et al performed another CyTOF analysis of *Salmonella* infection‐induced T‐cell responses and reconstructed the *Salmonella* serovar‐shaped repertoire of circulating effector CD4^+^ T cells.^120^ CyTOF also supported the investigations of immune factors associated with *Mycobacterium tuberculosis* ^121^, ^122^ and *Streptococcus pneumoniae* ^123^ infection.

Viral infections pose a constant challenge to the hosts’ immune system. Researchers have applied CyTOF to explore the immune alterations of patients infected with influenza,^124^ HIV,^125^, ^126^, ^127^, ^128^, ^129^, ^130^, ^131^, ^132^, ^133^ Japanese Encephalitis,^134^ Ebola,^135^ Gammaherpesvirus,^136^ chikungunya,^137^ hepatitis B,^138^, ^139^ as well as the mosquito‐borne human viral pathogens, including dengue^140^, ^141^, ^142^ and Zika,^143^, ^144^ and elucidated the fates of immune cells across viral infections. The use of CyTOF has also supported recent findings in COVID‐19 pathogenesis and immune perturbations,^145^, ^146^ where the results showed immunosuppression and dysfunction in PBMCs of COVID‐19‐infected patients. Leng et al investigated the inflammatory responses to SARS‐coronavirus‐2 (SARS‐COV‐2) in patients with COVID‐19 after treatment with ACE2‐mesenchymal stem cells.^147^ A key conclusion of this study is that treatment played a vital immune modulation role to reverse the functional failure of lymphocytes.

Some headway has also been made in employing CyTOF to investigate parasites‐induced immune regulatory networks. By profiling type‐2 immune response through a 37‐marker CyTOF analysis, Ruiter et al revealed detailed insights into the spectrum of immunomodulatory effects of helminth infection.^148^ Heathy Indonesians, Indonesians infected with soil‐transmitted helminths, and healthy Europeans that are not normally exposed to helminths were profiled. The profiling was conducted prior to and 1 year after deworming. The investigators found that immune signatures in Europeans and Indonesians were distinct and identified both Th2 and rare ILC2 cells, which expanded and acted as sources of type 2 cytokines in helminth‐infected patients. Only Th2 cells decreased after deworming, whereas the functional activity of both Th2 and ILC2s declined after anthelmintic treatment. Another study applied CyTOF to profile B‐cell compartments in *Plasmodium falciparum* malaria‐infected patients.^149^ In general, the understanding of immune subsets contributions in antibacterial, antiviral, and antiparasites defense by CyTOF profiling helps to define correlations of protective immune factors and guide effective vaccine development.

### 6.5 Cardiovascular disease

Atherosclerosis is affected by the interaction of pro‐ and anti‐inflammatory factors in the aorta. The immune system's heterogeneity provides an effective defense against various pathogens. The CyTOF technique helps to define the multifaceted contributions of immune defense within circulation and plaques (Table 4). Recently, Winkels's group demonstrated the phenotypic diversity of leukocytes from aortas of healthy and atherosclerotic mice via single‐cell RNA sequencing (scSeq) and CyTOF with a panel of 35 markers. The results revealed three principle B‐cell subsets that exhibited varied functional pathway responses, based on marker expressions of CD43 and CD220.^150^ Similarly, the Fernandez's group adopted 37‐plex CyTOF combined with scSeq and revealed a single‐cell immune landscape within human carotid artery plaques. Their analysis shows that, in symptomatic patients, plaques were characterized by distinct T‐cell subsets presenting markers of activation, differentiation, and exhaustion, whereas in plaques of asymptomatic patients, T cells and macrophages were activated and exhibited IL‐1β signaling.^151^ In essence, CyTOF technique is anticipated to accelerate discovery of underlying immunopathogenic factors for tailored cardiovascular immunotherapies.

### 6.6 Transplantation

CyTOF can also investigate the immune system perturbations after transplantation and patients’ heterogeneities in response to specific immunosuppressive regimes (Table 4).^152^, ^153^ One elegant implementation of CyTOF in transplantation research is the work of Fribourg et al^154^; through characterizing 26 kidney transplant recipients via serially collected PBMCs (before, 3 m, and 6 m after transplantation) using a panel of 35 immune markers, they defined a distinct role of T‐cell exhaustion in induction therapy responses and allograft functions. They delineated PD‐1^+^CD57^−^ exhausted T cells that correlated with better allograft function, which had low ATP production and cytokine secretion. This helps to identify T‐cell exhaustion‐associated factors for risk assessment. Allograft biopsies can be examined with the CyTOF technique at unprecedented resolution. CyTOF has also supported the identification of immune subsets that correlated with desensitization therapy results in patients undergoing sensitized kidney transplantation^155^ and a distinct immune profile that suggests operational tolerance in pediatric liver transplantation.^156^ In addition, CyTOF enables the investigations of protective and pathogenic immunity involved in transplants. As such, CyTOF analysis can link phenotype characteristics to variable posttransplant conditions, reveal the underlying mechanisms, and thus dramatically support the need to create and experiment with new strategies against posttransplant diseases.

### 6.7 Neurobiology and neuroimmune

The brain consists of highly dynamic and complex microenvironments that are populated with immune cells. The depiction of immune systems within the naïve or impaired brain compartment uncovers the diverse roles of immunomodulatory in central nervous system homeostasis. Korin et al characterized the immune system of the naïve mouse brain, with comparison to immune system in circulation, via CyTOF with 44 surface markers. The results show that most infiltrating leukocytes were mainly present at the brain boundaries, such as meninges and choroid plexus, and that CD44 distinguished resident and infiltrating immune cells.^157^ To better define human microglia cell repertoire, CyTOF was applied to investigate postmortem human microglia isolated from nine donors^158^ and interestingly revealed that mucin‐like hormone receptor 1 was expressed in human microglia, whereas it was not expressed in the monocytes and myeloid cells of circulation and cerebrospinal fluid. Researches also provided a nuanced comparison of the human brain's immune states during homeostasis, aging, and disease through the integration of CyTOF and other omic techniques and demonstrated that microglia subsets exhibited disease‐specific transformations in the brain (Table 4).^159^, ^160^, ^161^, ^162^, ^163^ Another interesting study focused on the impact of immune response to stroke on long‐term cognitive disability.^164^ With the approach of CyTOF, serial blood sample from patients was collected and analyzed over the course of a year to functionally and comprehensively characterize the immune response to stroke and its correlation with cognitive functioning between 90 and 365 days poststroke. The results confirmed a significant correlation between immune response measured during the stroke trajectories, which demonstrated the utility of CyTOF in the clinical prognosis and prediction of stroke. In summary, these studies depict the confounding utility of CyTOF in understanding immune orchestrations in human brain.

## 7 PERSPECTIVE

Recent progress and applications illuminate the salient features and the prospects of CyTOF in sketching the immune landscape. In essence, CyTOF can picture both innate^77^, ^137^, ^165^, ^166^ and adaptive^123^, ^167^ immune landscapes, which includes numerous phenotypically and functionally heterogeneous cell subsets of lymphoid and myeloid lineages that are involved in adequate surveillance and pathogens killing. Panoramic views of systemic immunity involving circulation and infiltration can also be obtained.^98^ In addition to horizontal comparison among groups of health and different disease statuses, CyTOF also supports longitudinal profiling,^164^ with the advancement in normalization^48^, ^168^ and batch effect minimizing.^49^, ^50^ CyTOF can also work in conjunction with other techniques including single‐cell genome and transcriptome profiling^169^, ^170^ and bioinformatic pipelines.^171^, ^172^ For instance, Zheng et al conducted CyTOF profiling of immune microenvironment in hepatocellular carcinoma and revealed that leading‐edge regions exhibited an increase of tumor‐associated CD4/CD8 double‐positive T (DPT) cells, which synergistically expressed PD‐1/HLA‐DR/ICOS/CD45RO.^173^ Single‐cell RNA‐seq was employed to characterize DPT cells and specifically identified PD‐1^high^ DPT cluster derived from intratumoral CD8^+^ T cells. Instead of only investigating single aspects of a cell subset, the joint analysis of multiple omics offers the opportunity to provide comprehensive insights into coordinated cellular process across different omic layers.

In general, the current progress made by CyTOF techniques mainly includes identifying cell ancestry to reveal the trajectory of cell differentiation and find reliable and specific immune signatures in patient stratification and treatment. CyTOF has facilitated the biospecimens analysis across large cohorts and permits the identification of ideal immunological biomarkers that are stable, reproducible, and easily measured, to reflect features underlying pathophysiology,^174^, ^175^ disease progression,^95^ or treatment schemes.^176^

We expect CyTOF to be established in pharmacological research and function as a powerful drug screening tool. Cell heterogeneities pose challenges for treatment selection.^177^ However, this heterogeneity is often obscured in conventional screening methods that yield average measurements of bulk populations, motivating the need for a high‐throughput single‐cell technique. CyTOF facilitates the screening of signaling network and dynamics of baseline or stimulated status. This creates a detailed response profile of the drug‐perturbed immune system and aids in the examination of drugs’ impact on the immune system.^178^ The understanding of drugs and its relationships with the immune system confers information for treating diseases, minimizing unwanted side effects, facilitates individual‐tailored medicine, and optimizes combination therapies.^179^ In addition, we expect CyTOF to enter clinical laboratories and gradually play a dominant role in immune compartment profiling. By virtue of its capacity of comprehensive and systematic immune characterization, CyTOF would play an indispensable role in routine evaluation of global health.

However, critical challenges remain. Several aspects of CyTOF require careful adaptation and routinization before its adaption in routine laboratory and clinical settings. Notable among these aspects are the standardization of single‐cell preparation techniques, rigorous antibody validation, sensitivity enhancement of antibody tags, a paradigm shift in pattern linking of data to relevant clinical outcomes, and even more parameters in routine use. Once the challenges in scalability, sensitivity, reproducibility, and reliability are overcome, it seems likely that in the near future, in tandem with its wider accessibility in pharmaceutical companies and clinical departments, CyTOF will assume the center stage to sketch immune landscape for varieties of diseases. We envision it playing a more confounding role in drug and vaccine development and immune demystification.

## 8 CONCLUSION

Researchers across biological fields have advanced the understanding of immunocyte heterogeneities involved in malignant diseases. By utilizing the increased dimensionality, CyTOF provides opportunities toward unlocking the mysteries surrounding immunopathogenesis and immune responses, which underlines clinical manifestation and shows immense potential for clinical laboratorial diagnosis, therapy efficacy monitoring, as well as treatment strategies. Ongoing improvements on technical aspects, including marker scalability and mass tag barcoding approaches for eliminating batch effects, facilitate the adaptation of CyTOF for routine applications in research and clinical laboratories. Coupled with innovative data analysis pipelines, application of CyTOF should accelerate the progress in fundamental and clinical immunology and advance the study and application of precision medicine.

## AUTHOR CONTRIBUTIONS

TZ and XD were involved in the conception, design, and drafting of the manuscript. TZ, AW, YL, and XD were involved in the editing and revision of the manuscript. All authors have read and approved the final manuscript.

## CONFLICT OF INTEREST

The authors declare no conflict of interest.
